# Molecular Epidemiology and Spatio-Temporal Dynamics of the H3N8 Equine Influenza Virus in South America

**DOI:** 10.3390/pathogens5040061

**Published:** 2016-10-16

**Authors:** Cecilia Olguin Perglione, Marcelo D. Golemba, Carolina Torres, Maria Barrandeguy

**Affiliations:** 1 Instituto de Virología CICVyA, Instituto Nacional de Tecnología Agropecuaria (INTA), Dr. Nicolás Repetto y De Los Reseros s/n Hurlingham B1686LQF, Buenos Aires, Argentina; olguin.cecilia@inta.gob.ar; 2 Hospital de Pediatría S.A.M.I.C. “Prof. Dr. Juan P. Garrahan”, Combate de los Pozos 1881, Ciudad Autónoma de Buenos Aires C1245AAM, Argentina; mgolemba@garrahan.gov.ar; 3 Cátedra de Virología, Facultad de Farmacia y Bioquímica, Universidad de Buenos Aires, Junín 956, Ciudad Autónoma de Buenos Aires C1113AAD, Argentina; ctorres@ffyb.uba.ar; 4 CONICET, Av. Rivadavia 1917, Ciudad Autónoma de Buenos Aires C1033AAJ, Argentina; 5 Escuela de Veterinaria, Universidad del Salvador, Viamonte 1856, Ciudad Autónoma de Buenos Aires C1056ABB, Argentina

**Keywords:** equine influenza, South America, haemagglutinin, phylodynamics, phylogeography

## Abstract

Equine influenza virus (EIV) is considered the most important respiratory pathogen of horses as outbreaks of the disease lead to substantial economic losses. The H3N8 EIV has caused respiratory disease in horses across the world, including South American countries. Nucleotide and deduced amino acid sequences for the complete haemagglutinin gene of the H3N8 EIV detected in South America since 1963 were analyzed. Phylogenetic and Bayesian coalescent analyses were carried out to study the origin, the time of the most recent common ancestors (tMRCA), the demographic and the phylogeographic patterns of the H3N8 EIV. The phylogenetic analysis demonstrated that the H3N8 EIV detected in South America grouped in 5 well-supported monophyletic clades, each associated with strains of different origins. The tMRCA estimated for each group suggested that the virus was circulating in North America at least one year before its effective circulation in the South American population. Phylogenetic and coalescent analyses revealed a polyphyletic behavior of the viruses causing the outbreaks in South America between 1963 and 2012, possibly due to the introduction of at least 4 different EIVs through the international movement of horses. In addition, phylodynamic analysis suggested South America as the starting point of the spread of the H3N8 EIV in 1963 and showed migration links from the United States to South America in the subsequent EIV irruptions. Further, an increase in the relative genetic diversity was observed between 2006 and 2007 and a subsequent decline since 2009, probably due to the co-circulation of different lineages and as a result of the incorporation of the Florida clade 2 strain in vaccines, respectively. The observed data highlight the importance of epidemiological surveillance and the implementation of appropriate quarantine procedures to prevent outbreaks of the disease.

## 1. Introduction

Equine influenza (EI) is one of the major infectious respiratory diseases of horses and is considered severely damaging in economic terms. This is due to the highly contagious nature of the virus which facilitates rapid spread among susceptible horses, with the consequent interruption of equestrian activities [1,2]. EI is usually a self-limiting disease characterized by pyrexia, coughing and nasal discharge [3]. Two subtypes of influenza A virus are known to infect horses, the H7N7 and the H3N8. The H7N7 was identified in 1956 during an outbreak of respiratory disease in horses in Czechoslovakia [4], and the H3N8 subtype was first detected in 1963 during a major epizootic of respiratory disease in horses in Florida, United States (USA) [5,6]. Both subtypes of viruses co-circulated for around 25 years; however, the H7N7 has not been isolated since 1979 and is considered to be extinct [7], and since that time, all outbreaks of EI worldwide have been caused by the H3N8 subtype.

Phylogenetic analysis of the equine influenza virus (EIV) haemagglutinin (HA) gene revealed that the H3N8 EIV evolved as a single lineage for at least two decades [8] and diverged during the mid-1980s, into the American and the European lineages, named according to the geographic origin of the isolates [9,10]. Subsequently, strains within the American lineage diverged into South American, Kentucky and Florida sub-lineages [11]. Nowadays, the Florida sub-lineage is predominant and has evolved into two antigenically different clades: Florida clade 1 and Florida clade 2 [9]. Florida clade 1 strains have been isolated in North America since 2003 [9] and have been associated with EI outbreaks in South Africa, Japan, Australia, Europe, Dubai and South America [1,12,13,14,15,16,17]. Florida clade 2 predominates in Europe, but has been also identified in some countries of Asia [18,19,20]. In addition to this evolutionary pattern, in a genome-scale analysis, Murcia et al. described that, according to the H3N8 EIV HA gene characteristics, 12 phylogenetically distinct clades (including Florida clade 1 and Florida clade 2) could be distinguished, each of them comprised by a group of viruses from a particular geographical region and sharing a common ancestor [21]. 

In South America, the first reported EI outbreak occurred in Chile in 1963 due to EIV the H3N8 infection [22,23]; a further occurrence, in 1977, was caused by the H7N7 subtype infection [24]. The H3N8 EIV re-emerged in Chile in several outbreaks that occurred in 1985 and 1992 [25,26]. Another episode of EI, which affected both vaccinated and unvaccinated horses, occurred in 2006, caused by a virus belonging to the H3N8 American lineage. The last reported outbreak of EI in Chile was in 2012 [27]. 

In Brazil, there has been evidence of EIV circulation since 1963 (A/eq/SaoPaulo/6/1963, GenBank accession number CY032293). Subsequent outbreaks of EIV occurred in 1969, 1976, 1986 and 1988 [28]. An extensive onset of acute respiratory disease in horses in 2012 was the last reported occurrence of EI in this country [29]. 

Regarding the EI situation in Uruguay, the extensive outbreak occurred in 2012 was the only one reported [16]. Nevertheless, previous evidence of the circulation of the virus is the HA gene nucleotide sequences of two strains available at the GenBank database, the H3N8, A/eq/Uruguay/1/1963 (GenBank accession number CY032421) and the H7N7, A/eq/Uruguay/1063/1976 (GenBank accession number CY036887).

With respect to the EI in Argentina, the first reported outbreak was in 1976 and the virus belonged to the H7N7 subtype [30]. Since then, all the reported EI outbreaks have been caused by the H3N8 subtype infection. The first detection occurred in the summer of 1985–1986, and is described as one of the major outbreaks of EI due to the rapid spread of the virus all across the country [31]. In 1993, a new episode of acute respiratory disease with high morbidity occurred among thoroughbred horses in Palermo’s and San Isidro’s racecourses [10,32]. Sporadic H3N8 EI virus incursions, with only a few horses involved, took place between 1994 and 2001 [11]. In 2005, EI arose among horses stabled in a jumping club in Buenos Aires city, which had temporarily received horses from Chile; the morbidity was low, not exceeding 10% of the horse population, and no cases outside this location were reported [33]. All the EIVs identified between 1993 and 2005 were grouped within the South American sub-lineage of the American lineage [11,33]. The last extensive outbreak of EI registered in Argentina was in 2012 [16].

The HA, one of the surface glycoproteins of the EIV, is the primary target of the protective immune response [34]. The HA1 domain contains the major neutralizing antibody binding sites identified from A to E [10,35]; if sufficient changes occur at these antigenic sites, the virus may evade antibody neutralization [9]. Vaccines provide protection through the stimulation of antibody response, especially against the HA glycoprotein [1,36], and vaccination plays a major role in controlling infection and disease, particularly for horses that travel widely, intermingle with other horses or take part in sports events [37]. Hence, the HA gene segment is of major importance and is the focus for EIV surveillance [34]. The World Organization for Animal Health OIE Expert Surveillance Panel (ESP) reviews the genetic, antigenic and epidemiological information of EI field outbreaks occurring worldwide, in order to evaluate the vaccine effectiveness and elaborate recommendations to ensure that the vaccines contain epidemiologically relevant strains [36].

In spite of the fact that viral surveillance and molecular descriptions have been performed on EIV, no studies have analyzed the origin and the spatio-temporal phylodynamics of EIV in South America. Thus, the aim of this work was to describe the molecular characteristics of the H3N8 EIV detected in South America since its first identification, to infer the potential source of infections and to study its demographic and phylogeographic patterns.

## 2. Results

### 2.1. Phylogenetic Analysis 

**Figure 1:**
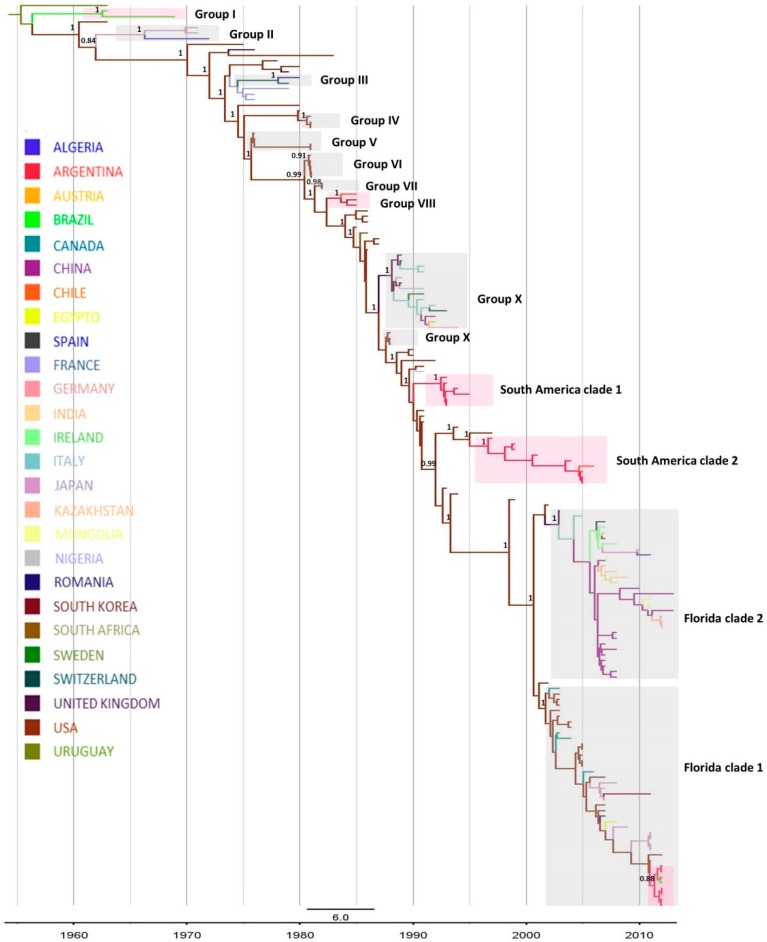
Maximum clade credibility tree for the complete HA gene of the H3N8 EIV. The branches are in time scale (years) and are colored on the basis of the most probable ancestor location. Posterior values for relevant groups are shown at nodes. Groups highlighted in Magenta correspond to South American clades.

EIV HA gene phylogenetic analyses were carried out with two different data sets: (a) partial HA1 and (b) complete HA nucleotide sequence gene. The topology of the trees obtained with maximum likelihood (ML) was similar for both data sets (Figures S1 and S2). In addition, the time-scaled maximum clade credibility (MCC) tree inferred by Bayesian coalescent analysis also displayed a similar phylogenetic pattern (Figure 1) [38]. The resulting trees grouped all the viruses included in this study into five well-supported clusters: Pre-divergent, Eurasian, American and Florida clade 1 and Florida clade 2 sub-lineages (Figure 1, Figures S1 and S2). Following the criteria introduced by Murcia, the twelve described different clades could also be distinguished in the trees obtained with the complete HA date set [21]. However, three of the twelve groups (VI, VII and IX) were not observed in the ML tree obtained with the partial HA data set, probably due to a lower phylogenetic signal in the partial HA data set compared to the complete HA data set. Moreover, two additional clades, called South America clade 1 and South American clade 2, were found in all the trees. Correlating, isolates from Groups I to VIII belong to the Pre-divergent lineage, whereas Group IX and X belong to the American and the Eurasian lineage, respectively. 

All the H3N8 EIVs detected in South America were grouped into five monophyletic clades, sustained by high support values (Figure 1, Figures S1 and S2). As previously described by Murcia et al. [21], Group I is made up of strains detected in Brazil in 1963 and 1969 (*n* = 2), and Group VIII of strains from Argentina (*n* = 1), Chile (*n* = 1) and interestingly, from USA (*n* = 1) in 1985; these two groups were included in the Pre-divergent lineage. The EIV detected from 1993 to 1996 was grouped in the South American clade 1, which is composed only of Argentinian strains (*n* = 6). The EIV identified between 1997 and 2006 belonged to the South American clade 2, grouping strains detected in Argentina in 1997, 2001, 2004 and 2005 (*n* = 8) and in Chile in 2006 (*n* = 1). The South American clades 1 and 2 were included in the American lineage constituting two separate groups, independent from Murcia’s Group X. The EIV detected in Chile (*n* = 1), Brazil, (*n* = 1), Uruguay (*n* = 1) and Argentina (*n* = 6) in 2012 belonged to the Florida clade 1 lineage (Figure 1, Figures S1 and S2).

### 2.2. Phylodynamic Analysis

A Bayesian coalescent analysis of the complete HA gene was carried out to study the demographic and phylogeographic pattern of EIV, and particularly, to estimate the ancestral times associated with South American viruses. The time of the most recent common ancestors (tMRCA) was estimated for the different groups: Group I, 1962; Group VIII, 1984; South American clade 1, 1992 and South American clade 2, 1997. As for the viruses detected in South America in 2012, which grouped within the Florida clade 1, the tMRCA was estimated in 2011 (Table 1) [38].

In addition, the geographic spread pattern of the H3N8 EIV in South America was studied. The most probable ancestral locations of different groups were estimated and are represented in different colors in the MCC tree (Figure 1). Particularly, despite the fact that the analysis estimated Brazil as the most probable ancestral location of the strains belonging to Group I (*p* = 0.98), the location of the ancestral virus originating this group could not be defined with certainty. For Group VIII, the most probable origin appears to be USA (*p* = 0.97). The South American clade 1 EIV, identified between 1993 and 1996, would derive from strains circulating in USA (*p* = 1). The most probable ancestral location of South American clade 2 and Florida clade 1 was also USA, with high probabilities (*p* = 0.98 and *p* = 0.97, respectively). In accordance with this observation, the South American clade 2 was closely related to A/eq/California/4537/97 and A/eq/Kentucky/1/94 strains, while Florida clade 1 strains from South America were closely related to A/eq/Florida/146609/11 strain; these were identified as probable ancestors of these groups. 

**Figure 2:**
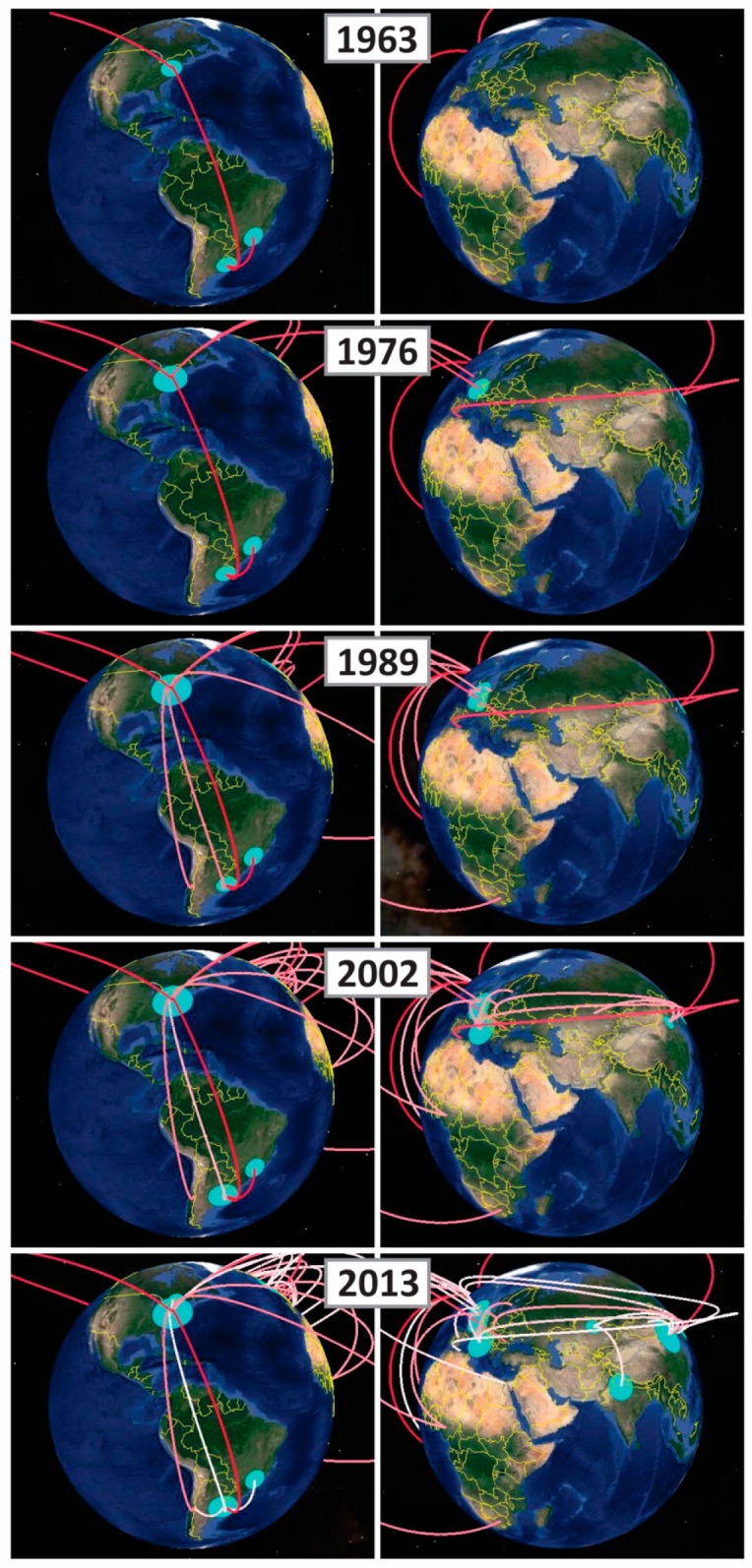
Spatio-temporal diffusion process for EIV in America (left side) and Europe, Asia and Africa (right side). Lines represent the maximum clade credibility tree projected on the surface. The red-white colour gradient represents the relative age of the dispersal pattern (older-recent). The size of light blue polygons represents lineage density for each location.

To gain insight into the spatio-temporal dynamics of the viral diffusion process, the MCC tree can be visualized over time (Figure 2). The earliest divergence events were observed from Uruguay to USA and Brazil between 1963 and 1969. After that, in 1971, the virus spread from USA into Asia and subsequently from Asia into Africa in 1972. In 1973, the H3N8 EIV entered from USA into France, and during the next few years the virus has spread within the European continent. The first link from USA to South America occurred between 1983 and 1985, when the virus spread into Argentina and Chile. Later, the EIV spread from USA into South Africa in 1986, and circulated in Europe during the period 1989 and 1992. A new entrance from USA into Africa occurred in 1991 and from Europe into Asia in 1992. A second migration wave from USA to South America was inferred between 1991 and 1993, and the virus was identified in Argentina in 1993. A third migration link between USA and South America was inferred in 1996 and after its introduction, the virus spread across Argentina (A/eq/Argentine/E-433/1997) and Chile (A/equine/Lonquen/1/2006). Between 2002 and 2007, new introductions from USA to Europe and Asia occurred, and subsequently from Asia into Europe. The last migration link between USA and South America was observed in 2011, after which the virus circulated in Uruguay, Argentina and Brazil (Figure 2).

**Figure 3:**
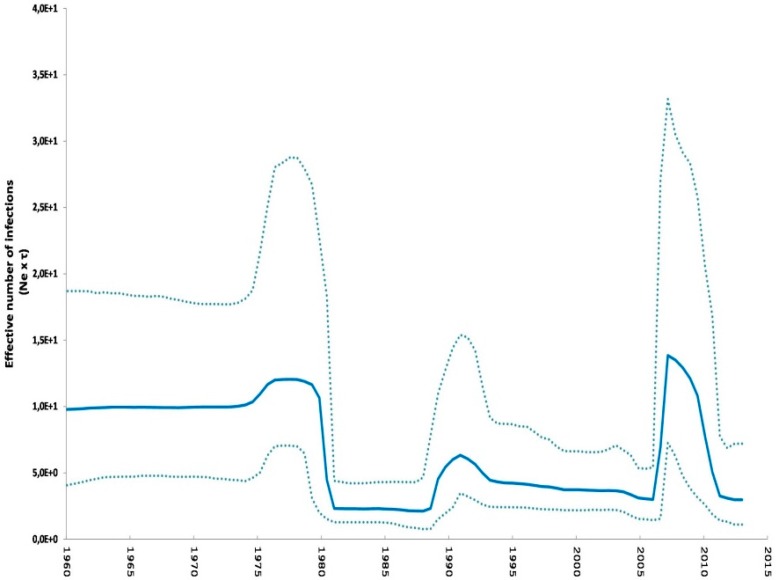
Phylodynamic reconstruction for complete HA data set showing the relative genetic diversity through time (Ne x t). The thick solid line represents the mean value and the dash lines the HPD 95% values.

In addition, demographic reconstruction revealed that genetic diversity has remained constant between 1960 and 1970, followed by an abrupt drop in the early 1980s, and an increase and stabilization from about 1990 onwards. In 2006, viral diversity shows a remarkable increase with a maximum in 2007, and then a slight decrease from 2007 to 2009 followed by an abrupt decrease from then on (Figure 3) [38]. 

### 2.3. Amino Acid Alignment

**Figure 4:**
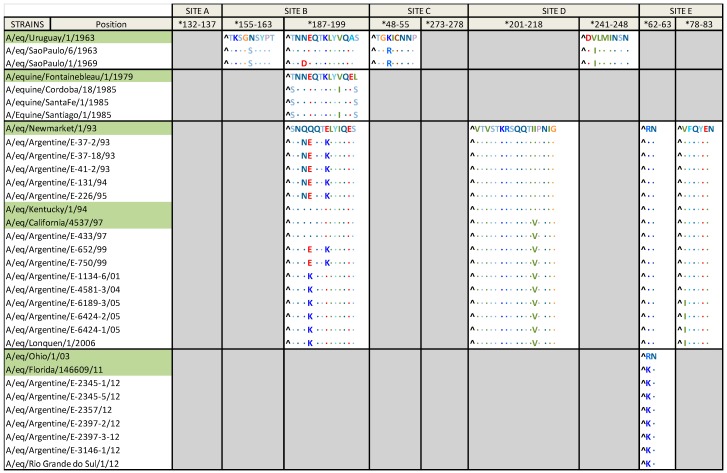
Amino acid changes in the antigenic sites.

The HA1 derived amino acid (aa) sequences from the South American strains were aligned with the prototype and the inferred ancestor strains of each group (Figure 4 and Table 2).

Strains belonging to Group I were compared with A/eq/Uruguay/1/1963, as the H3N8 EIV is suggested to have originated in South America. Eleven aa substitutions were found, three of them at antigenic sites: K50R (site C), which is exclusive to this group, N159S (site B) and V242I (site D). Three additional aa changes, all of them outside of antigenic sites and exclusive to this group, were observed: T131N, S149N and S265G. The remaining aa substitutions were: G6S, G7D, N291E, Q327R and I328L, which are also found in other strains. The strain detected in Sao Paulo in 1969 possesses an additional aa substitution at the antigenic site B (N189D), which is unique to this strain. The aa G and R at positions 82 and 323 respectively, are observed in Group I and Uruguay/1/1963 but not in other H3N8 EIV strains.

Compared with A/eq/Fontainebleau/79 (Pre-divergent prototype strain), the strains belonging to Group VIII identified in South America presented seven aa substitutions: T46I, A93T, R140K, V223I, I267V, T187S and L199S, the last two at the antigenic site B.

In comparison with A/eq/Newmarket/1/93 (American lineage prototype strain), viruses within the South American clade 1 carried four aa substitutions at the HA1, three of them at the antigenic site B: Q189N, Q190E and E193K. The remaining aa substitution, N312K, is present only in the strains of this lineage. The South American clade 2 strains showed three aa substitutions; one of these substitutions, I214V, is located at the antigenic site D and is also present in one of the possible ancestors, A/eq/California/4537/97. The remaining aa changes are S92N, which is exclusive to this group, and D104N. In addition to the described aa substitutions, the strains detected in 1999 possess the two aa changes at antigenic site B, Q190E and E193K, which were also observed in strains from the South American clade 1. The EIV strains detected between 2001 and 2006 have evolved with a new aa change, Q190K, in the antigenic site B, which is also exclusive to this group. The strains detected in 2005 and 2006 possess an additional and distinctive aa change, V78I.

The aa substitutions presented in the Uruguayan, Brazilian and Argentinian strains detected in 2012, when compared with the Florida clade 1 prototype strain (A/eq/Ohio/01/03) are: G7D, R62K (in antigenic site E), D104N, A138S and V223I, which are also present in the possible origin of the group, A/eq/Florida/146609/11 strain. In addition, two different sub-populations can be recognized among them, one with a substitution at K-14A at the predicted peptide signal sequence; and the other one at K-14T also at the predicted peptide signal sequence and M70V, the latter being present only in Argentinian strains. With the available data for the strain detected in Chile in 2012 (deduced aa sequence from position 1 to 159), the same four aa substitutions can be observed. Except for K-14A and M70V, the Florida clade 1 South American strains have the same aa substitutions as A/eq/Florida/146609/11.

## 3. Discussion

Since 1963, the EIV H3N8 subtype has been the cause of numerous outbreaks of respiratory disease in horses worldwide, including South American countries where the horse industry is highly significant. In this study, comprehensive phylogenetic and phylodynamic analyses of the EIV South American strains were carried out to study their diversification, to infer their origin and to estimate the dynamics of their geographical spread. This information could contribute to a better understanding of the epidemiology of the H3N8 EIV infections worldwide and particularly in South America, and could also allow Animal Health Authorities to improve the preventive and control measures currently in use.

The spatio-temporal analysis demonstrated that the H3N8 EIV strains detected in different outbreaks of acute respiratory disease in horses in South America were grouped into five well supported monophyletic clades. As was previously described, Group I is the oldest clade, and is made up of strains detected between 1963 and 1969 also grouped into the Pre-divergent lineage. It is believed that EIV of the H3N8 subtype crossed the species barrier from birds in the early 1960s and has subsequently spread worldwide [1,3,36,39]. It has been suggested that the H3N8 EIV subtype originated in South America, since it was identified after the arrival of thoroughbred horses imported from Argentina to Miami in 1963 [5,6]. Even though there are no reports of EIV circulation in Argentina at that time, there is evidence of H3N8 EIV infection and disease in horses in Chile, Brazil and Uruguay [22,23]. Group VIII, also in the Pre-divergent lineage, consists of strains circulating in 1985 [21], which comprise the first isolation of an H3N8 EIV in Argentina [31] and a reintroduction of this virus in Chile, apparently after the entry of horses from Argentina [25,26]. Surprisingly, a strain detected in USA in 1985 belonged to this group, which could be due, as in 1963, to subclinically infected horses traveling from South America to USA. The South America clade 1, described by Lai in 2001 [11], groups only Argentinian strains. This lineage circulated for a period of 3 years (between 1993 and 1996) and it has not been detected since that time. The South American clade 2, that was previously reported by Miño et al. [33], includes Argentinian and Chilean EIV strains detected between 1997 and 2006, and it has been previously suggested that based on the close resemblance of the viruses, it entered Chile from Argentina [22]. The South American clade 1 and 2 are clustered into the American lineage. These clades are made up of strains that circulated exclusively in South America between 1993 and 2006; yet, the clades would have different ancestors. Finally, the causative virus of the multiple large-scale outbreak of EI in 2012 in South America belongs to the Florida clade 1, and spread among several countries, yielding important economic losses [1,16,29]. In addition, the same virus was also detected in Dubai in 2012, in an outbreak in a quarantine facility after the entry of a group of endurance horses from Uruguay [1]. 

The phylogenetic patterns of EIVs identified in South America are consistent with previous studies that show that viruses from the same lineage are usually isolated in a particular geographical region [11,21]. Interestingly, the viruses of each clade detected in South America have circulated only for a few years, with no evidence of reemergence. This could be interpreted as a consequence of the development of wide population immunity due to natural infections and/or efficacious vaccination programs. The polyphyletic behavior of the viruses causing the outbreaks of equine influenza, which took place in South America between 1963 and 2012, seem to be due to the introduction of at least four different viruses, probably through the movement of subclinically infected horses. These findings are in agreement with previous consideration that incursion of the influenza virus into new equine populations can occur through subclinically infected horses facilitated by short quarantine periods when horses travel internationally for competitions or sales [10,34]. 

Although the complete HA data set included all the HA complete sequences available at the Influenza Research Database (*n* = 144), the most related sequences of the Group I, Group VIII and South America clade 1 could not be estimated. Possibly, the analysis of other genome segments could bring up more information and help to estimate the most related strains of these old South American viruses. Additionally, the spatial and temporal dynamics for the geographic spread provided more information on the migration patterns of the H3N8 EIV. The ancestral location for Group I could not be determined with confidence; this could be due to a possible biased sampling of the EIV H3N8 during that period for which few isolates were sequenced. Despite being unable to accurately infer the possible ancestors for Group VIII and South America clade 1, migration links were established from USA to South America, so there is a high probability of the former being the ancestral location of these groups, as well as of South America clade 2 and Florida clade 1. In agreement with previous reports [5,6], the discrete phylogeographic model suggested South America as the starting point of the spread of the EIV H3N8. In the map, migration links were established repeatedly between North and South America, which is consistent with the frequent movement of horses that exists between USA and South America and vice versa. Thoroughbred shuttle stallions, for example, travel from USA to South America every breeding season and several thoroughbred mares are bought for genetic improvement every year; furthermore, polo horses travel from Argentina for the polo season in USA, coming back to Argentina when the season is over. Additionally, the diffusion process also suggested USA as an important primary origin for the spread of the EIV H3N8 into Asia and Europe. This is consistent with a report of the first detection of this virus in Europe, in an important outbreak of EI in Switzerland in 1965, which was considered a major transcontinental pandemic of the EIV H3N8 [21,40]. However, a sub notification of EIV outbreaks could exist in other countries that could act as important spreaders of the virus. The tMRCA estimated for the five clades detected in South America suggests that the ancestor strains had been circulating in the Northern hemisphere at least one year before their effective circulation in South America, with the exception of Miami 63, that apparently moved from South America to North America [5,6].

As an aid in the understanding of the evolutionary pathways and population dynamics of the H3N8 EIV, a demographic reconstruction was used to examine the changes of the effective number of viral population size. The relative genetic diversity observed between 1963 and 2008 was similar to that previously described by Murcia et al. [21]. In our work, the analyzed time was extended from 2008 onwards. Since 2006, an accelerated increase in the relative genetic diversity was observed, with a peak in 2007, followed by an abrupt decline in 2009. The co-circulation of the South American clade 2, Florida clade 1 and Florida clade 2 sub-lineages strains [41] could be the reason of the observed increase in the relative genetic diversity. The decrease observed since 2009 could have resulted from the introduction of the Florida clade 2 strain in the vaccines, as recommended by the ESP [42]. However, not all the manufacturers have updated their vaccines to contain Florida clade 2 strain. For this reason, another possible explanation may be the lack of active surveillance in some regions of the globe, or the lack of sequences being made publicly available. The limited viral diversity found in the H3N8 EIV strains is noteworthy, and it was homologated to the one observed with Influenza B virus in humans, with only two lineages circulating [11,34].

Multiple amino acid substitutions have occurred in the HA of the viruses detected in South America since the first description in 1963. The strains belonging to the Group I, Group VIII, South American clade 1 and South American clade 2, have aa changes at antigenic site B compared with the prototype strains. This observation is important because the fact that antigenic site B is positioned at the top of HA1 implies that aa changes occurring in this site could compromise the viral antigenicity [43,44]. In addition, some amino acid substitutions such as N312K and S92N present in the South American clade 1 and 2, respectively, seem to be exclusive to South American strains. These aas could be hence used as molecular markers to identify the reemergence of those viruses in the future. On the contrary, no aa changes in the antigenic site B were observed in Florida clade 1 strains detected in South America in 2012 when comparing it with the prototype strain A/eq/Ohio/2003. Nevertheless, two subpopulations characterized by the aa substitutions K-14A and M70V (not in antigenic sites) were observed indicating the occurrence of some degree of evolutionary change. These aa changes are only found in South American strains, and M70V was observed only in Argentinian strains [16]. Nevertheless, these changes, outside the antigenic sites, do not imply the necessity to replace the vaccine strain in regard to the Florida clade 1 strain, A/eq/Ohio/2003, recommended by the ESP. This is additionally supported by the antigenic analysis carried out with 2012 Brazilian and Argentinian isolates, that showed a good recognition by sera raised against other members of Florida clade 1 [16,29]. It is important to highlight that at the time of the last outbreak of EI in South America, the vaccines on the market were not in line with the ESP recommendations; fortunately, after the outbreak many vaccine manufacturers in Uruguay and Argentina updated their vaccine strains [16,29].

## 4. Experimental Section

### 4.1. HA Gene Sequencing

The complete HA nucleotide sequence data of 19 viruses detected in different outbreaks of EI in Argentina during the period 1993 and 2012, and one strain detected in Uruguay in 2012, were obtained (Table S1). The sequence data of 13 of these viruses were derived from RNA extracted from nasopharyngeal swabs; the remaining 7 viruses (A/eq/Argentine/E131/1994, A/eq/Argentine/E226/1995, A/eq/Argentine/E433/1997, A/eq/Argentine/E652/1999, A/eq/Argentine/E750/1999, A/eq/Argentine/E1134-7/2001 and A/eq/Argentine/E6189-3/2005) required amplification in embryonated hen’s eggs prior to sequencing. 

The complete HA gene was amplified in two separate reactions by the OneStep RT-PCR kit (Qiagen) using primers previously described by Gildea et al. [12]. The PCR products were purified using the ExoStar (Illustra™, GE Healthcare UK Limited, Amersham, UK), according to manufacturer’s recommendations, and sequenced by the “Unidad de Genómica, Instituto de Biotecnología, INTA, Hurlingham”. 

### 4.2. Sequence Analysis and Phylogenetic Trees

The obtained HA nucleotide sequences were edited with the BioEdit software v7.0.9.0 [45] and were aligned using ClustalW in the software package of BioEdit, along with 144 reference HA complete sequences (complete HA data set) available at the Influenza Research database (IRD) up to January 2015 and one sequence from Brazil obtained from the GISAID EpiFlu™ Database. In addition, a partial HA1 nucleotide sequence (from position 52 to 545) from a strain detected in Chile in 2012 available at the IRD, was also analyzed in the partial HA1 data set (Table S2).

Phylogenetic trees for partial HA1 and complete HA nucleotide sequences were inferred by the maximum likelihood (ML) method using PhyML v3.1 software [46]. The best fit evolutionary models of nucleotide substitution were estimated according to the Akaike Information Criterion (AIC) statistics obtained with the jModelTest v2.1.6 software [47]. Branch connecting sequence A/equine/Uruguay/1/1963 detected in Uruguay in 1963 with the others was selected for rooting the phylogenetic trees inferred from both data sets.

### 4.3. Phylodynamic Analysis 

The nucleotide sequences including the information about location and the year of isolation from the complete HA data set were introduced in the Bayesian coalescent analysis to estimate the time of the most recent common ancestor (tMRCA), the population dynamics and the spatio-temporal diffusion process. The analysis was carried out using the best fit evolutionary model of nucleotide substitution (see above), the Bayesian Skyline Plot (BSP) model and the relaxed uncorrelated lognormal molecular clock. A uniform prior to nucleotide substitution rate from 1 × 10^−3^ to 3 × 10^−3^ s/s/year was used to carry out the analysis, which included the nucleotide substitution rate for HA gene of the EIV H3N8 previously calculated by Murcia et al. [21], corresponding to 1.7 × 10^−3^ s/s/year. To infer the ancestor location and viral migration events, we grouped the H3N8 isolates into 26 states (countries) using the discrete phylogeographic asymmetric model implemented in BEAST v1.8.3 software [48], which uses a continuous time Markov Chain (CTMC) over discrete sampling locations and allows different rates of diffusion between each pair of locations [49] Analyses were run using BEAGLE library [50,51] at the CIPRES Science Gateway server [52]. The convergence of the parameters to a stationary distribution was assessed with the TRACER v1.6 software [53]. Two independent analyses were performed and results were combined. The trees were summarized in a maximum clade credibility (MCC) tree with temporal and spatial annotation using the Tree Annotator v1.8.3 software after discarding the burn-in samples. To visualize the geographic migration of the virus over the time, a keyhole markup language (KML) file was generated using SpreaD3 v0.9.6 [54], which can be visualized via Google Earth V. Monophyly was constrained for all sequences except for sequence A/eq/Uruguay/1/1963 detected in Uruguay in 1963.

### 4.4. Amino Acid Analysis 

The deduced amino acid sequences of HA1 were aligned with representative strains and the most related sequences of each group. Alignments were analyzed using the BioEdit v7.0.9.0 software [45]. 

## 5. Conclusions

As a conclusion, EIV outbreaks that have taken place in South American countries were caused by at least four different and independent introductions of the virus, presumably brought in through the international movement of horses. Afterwards, these viruses circulated and induced outbreaks of acute respiratory disease at a regional level, as the exchange of horses for competition amongst South American countries is extremely frequent. Unfortunately, there is no information available related to EIV infections in other South American countries; thus, we may infer that either the disease has not occurred, or there are no surveillance programs, or there is an unavailability of diagnostic laboratories or a sub-notification of EIV outbreaks, or a combination of these. Finally, taking into account that the spread of EIV worldwide is facilitated by the international movement of horses, adequate epidemiological surveillance programs, appropriate quarantine procedures and the maintenance of high levels of population immunity through vaccination with updated and efficacious vaccines, are critical aspects in the control of EI. 

## Figures and Tables

**Table 1:** Estimated time of the most recent common ancestor (tMRCA) for the five clades detected in South America by Bayesian Coalescent analysis.

Clade	Period	tMRCA (HPD 95%) *
Group I	1963–1969	1962 (1961–1963)
Group VIII	1985	1984 (1983–1984)
South American Clade 1	1993–1996	1992 (1992–1993)
South American Clade 2	1997–2005	1997 (1996–1997)
Florida Clade 1	2012	2011 (2011–2012)

* HPD95%: Highest posterior density interval (95%).

**Table 2:** Amino acid substitution characterizing the South American groups.

Group	Aa Substitution
Group I	K50R; T131N; S149N; S265G; L431I; A476T; L496V
Group I and A/eq/Uruguay/1/1963	G82; R323; I347; G381
South American clade 1	N312K
South American clade 2	S92N
Florida clade 1	K-14A; M70V

## Supplementary Materials

The following are available online at www.mdpi.com/2076-0817/5/4/61/s1, Figure S1: Maximum likelihood phylogenetic tree for the Partial HA 1 gene. Figure S2: Maximum likelihood phylogenetic tree for the Complete HA gene. Table S1: Outbreaks of EIV in Argentina between 1993 and 2012. Table S2: Equine influenza virus included in Phylogenetic and Coalescent analysis.
