# Supplementary material for: Molecular Epidemiology and Spatio-Temporal Dynamics of the H3N8 Equine Influenza Virus in South America
Source: Pathogens. 2016 Oct 16;5(4):61. doi: 10.3390/pathogens5040061 (PMC5198161; doi:10.3390/pathogens5040061)
Supplement: Supplementary file 1 [file pathogens-05-00061-s001.docx]

Supplementary Materials: Molecular Epidemiology and Spatio-Temporal Dynamics of H3N8 Equine Influenza Virus in South America

Cecilia Olguin Perglione, Marcelo D. Golemba, Carolina Torres and Maria Barrandeguy


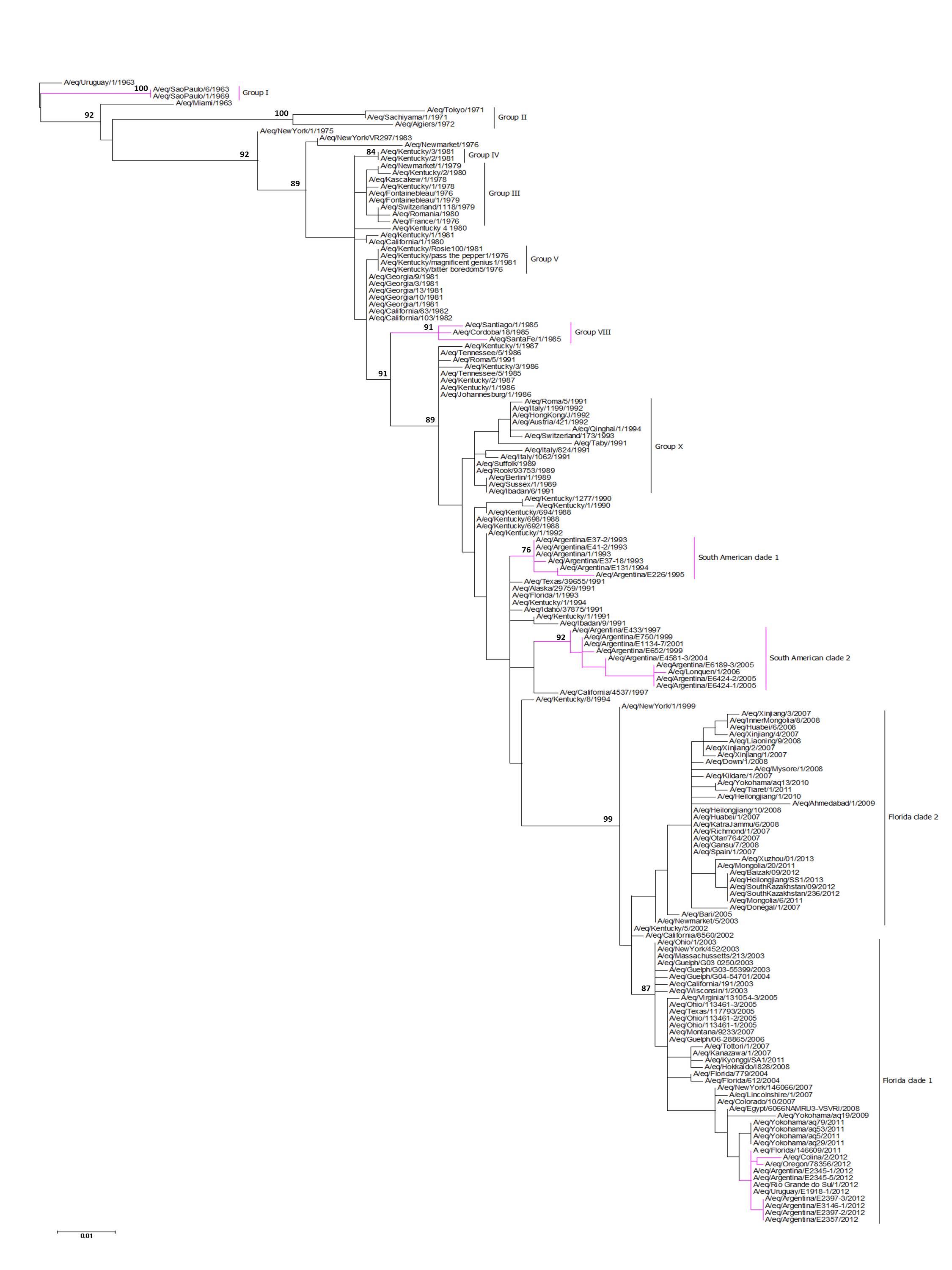


**Figure S1.** Maximum likelihood phylogenetic tree for the Partial HA 1 gene: Bootstrap values higher than 70% are shown at nodes for relevant groups. Magenta branches correspond to South American clades.


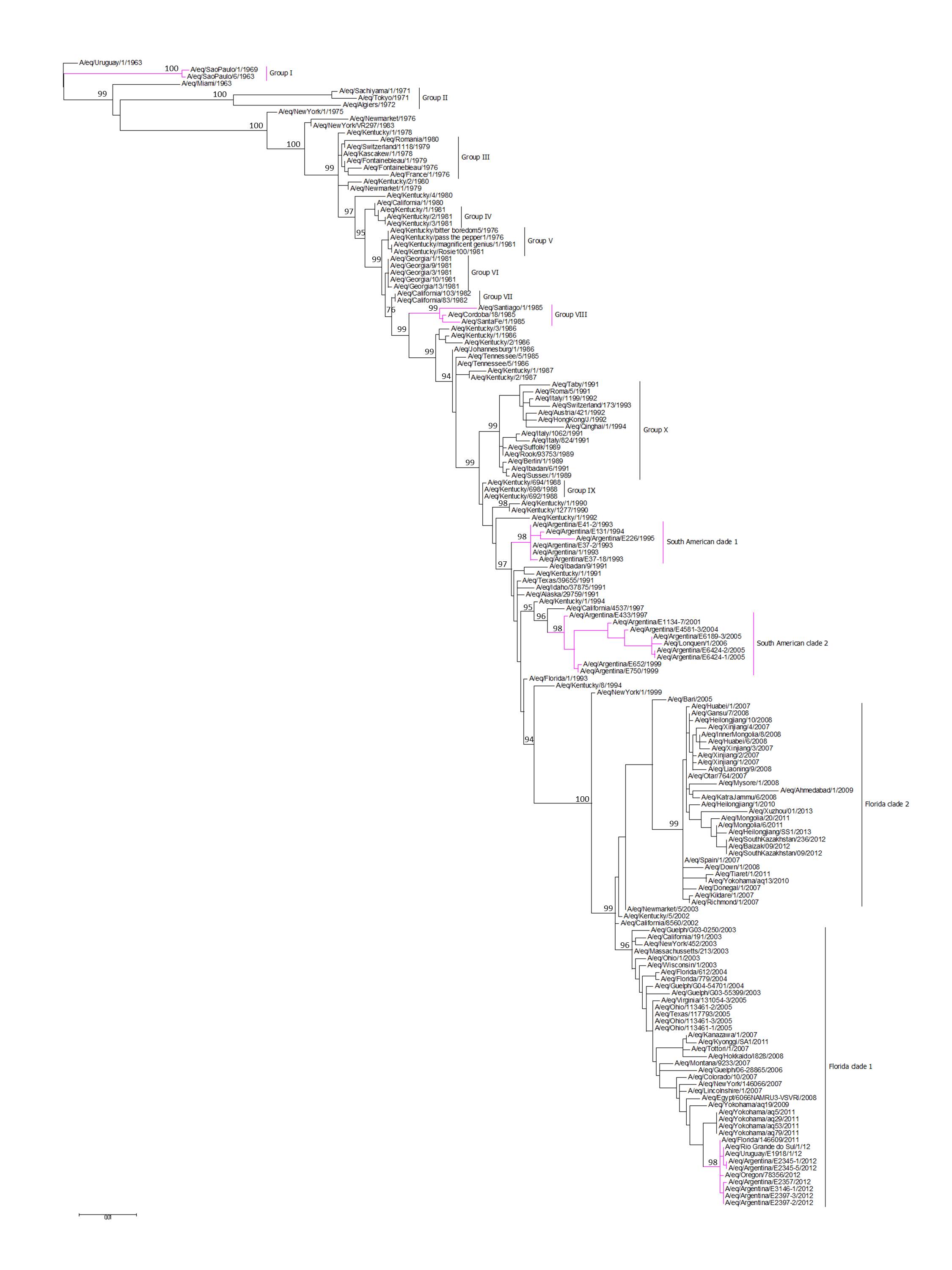


**Figure S2.** Maximum likelihood phylogenetic tree for the Complete HA gene: Bootstrap values higher than 70% are shown at nodes for relevant groups. Magenta branches correspond to South American clades.

**Table S1.** Outbreaks of EIV in Argentina between 1993 and 2012.

| **Date of Outbreak** | **Location** | **Type of Premise** | **Virus Name** | **HA acc N°** |
| --- | --- | --- | --- | --- |
| March 1993 | Buenos Aires city, Argentina | Racing and training yard | A/eq/Argentine/E37-2/93 | KX815358 |
| March 1993 | Buenos Aires city, Argentina | TB breeding farm | A/eq/Argentine/E37-18/93 | KX815359 |
| March 1993 | Buenos Aires city, Argentina | Racing and training yard | A/eq/Argentine/E41-2/93 | KX815360 |
| May 1994 | Buenos Aires city, Argentina | Jumping horse farm | A/eq/Argentine/E131/94 | KX815361 |
| June 1995 | San Isidro, Buenos Aires province, Argentina | Racing and training yard | A/eq/Argentine/E226/1995 | KX815362 |
| May 1997 | Pilar, Buenos Aires province, Argentina | TB breeding farm | A/eq/Argentine/E433/1997 | KX815363 |
| February 1999 | Lincoln, Buenos Aires province, Argentina | Embrio transfer center | A/eq/Argentine/E652/1999 | KX815364 |
| September 1999 | Mar del Plata, Buenos Aires province, Argentina | TB breeding farm | A/eq/Argentine/E750/1999 | KX815365 |
| June 2001 | Buenos Aires city, Argentina | Rural fair | A/eq/Argentine/E1134-7/2001 | KX815366 |
| October 2004 | Buenos Aires city, Argentina | Riding school | A/eq/Argentine/E4581-3/2004 | KX815372 |
| August 2005 | Chascomus, Buenos Aires province, Argentina | Jumping horse farm | A/eq/Argentine/E6189-3/2005 | KX815373 |
| October 2005 | San Isidro, Buenos Aires province, Argentina | Racing and training yard | A/eq/Argentine/E6424-1/2005 | KX815375 |
| October 2005 | San Isidro, Buenos Aires province, Argentina | Racing and training yard | A/eq/Argentine/E6424-2/2005 | KX815374 |
| March 2012 | Montevideo, Uruguay | Racing and training yard | A/eq/Uruguay/E1918-1/2012 | KX815377 |
| July 2012 | Buenos Aires city, Argentina | Racing and training yard | A/eq/Argentine/E2345-1/2012 | KX815367 |
| July 2012 | Buenos Aires city, Argentina | Racing and training yard | A/eq/Argentine/E2345-5/2012 | KX815368 |
| July 2012 | Buenos Aires city, Argentina | Racing and training yard | A/eq/Argentine/E2357/2012 | KX815369 |
| July 2012 | San Antonio de Areco, Buenos Aires province, Argentina | Racing and training yard | A/eq/Argentine/E2397-2/2012 | KX815370 |
| July 2012 | San Antonio de Areco, Buenos Aires province, Argentina | TB breeding farm | A/eq/Argentine/E2397-3/2012 | KX815376 |
| July 2012 | Esquel, Chubut province, Argentina | Show horses | A/eq/Argentine/E3146-1/2012 | KX815371 |

TB = Thoroughbred; HA acc N° = HA GenBank accession number.

**Table S2.** Equine influenza virus included in Phylogenetic and Coalescent analysis.

| **Location** | **Strain Name** | **Accession Number** |
| --- | --- | --- |
| India | A/equine/Ahmedabad/1/2009 | GU396102 |
| USA | A/equine/Alaska/29759/1991 | CY030157 |
| Algeria | A/equine/Algiers/1/1972 | CY032945 |
| Argentina | A/equine/Argentina/1/93 | L39913 |
| Austria | A/equine/Austria/421/1992 | CY032349 |
| Kazakhstan | A/equine/Baizak/09/2012 | KP202377 |
| Italy | A/equine/Bari/2005 | EF117330 |
| Germany | A/equine/Berlin/1/1989 | CY032413 |
| USA | A/equine/California/1/1980 | CY028812 |
| USA | A/equine/California/103/1982 | CY028908 |
| USA | A/equine/California/191/2003 | DQ124195.1 |
| USA | A/equine/California/4537/1997 | CY030751 |
| USA | A/equine/California/83/1982 | CY030085 |
| USA | A/equine/California/8560/2002 | CY030759 |
| USA | A/equine/Colorado/10/2007 | [HQ917682](http://www.ncbi.nlm.nih.gov/entrez/viewer.fcgi??db=nucleotide&val=HQ917682) |
| Argentina | A/equine/Cordoba/18/1985 | CY032301 |
| Ireland | A/equine/Donegal/1/2007 | JN222934 |
| Ireland | A/equine/Down/1/2008 | JN222937 |
| Egypt | A/equine/Egypt/6066NAMRU3-VSVRI/2008 | FJ209731 |
| USA | A/equine/FL/146609/2011 | KM359811 |
| USA | A/equine/Florida/1/93 | L39916 |
| USA | A/equine/Florida/612/2004 | [JF498588](http://www.ncbi.nlm.nih.gov/entrez/viewer.fcgi??db=nucleotide&val=JF498588) |
| USA | A/equine/Florida/779/2004 | [JF498589](http://www.ncbi.nlm.nih.gov/entrez/viewer.fcgi??db=nucleotide&val=JF498589) |
| France | A/equine/Fontainebleau/76 | [M24723](http://www.ncbi.nlm.nih.gov/entrez/viewer.fcgi??db=nucleotide&val=M24723) |
| France | A/equine/Fontainbleu/1/1979 | CY032405 |
| France | A/equine/France/1/1976 | M73773 |
| China | A/equine/Gansu/7/2008 | EU794495 |
| USA | A/equine/Georgia/1/1981 | CY028860 |
| USA | A/equine/Georgia/10/1981 | CY028884 |
| USA | A/equine/Georgia/13/1981 | CY028892 |
| USA | A/equine/Georgia/3/1981 | CY028868 |
| USA | A/equine/Georgia/9/1981 | CY028876 |
| Canada | A/equine/Guelph/06-28865/2006 | EU855744 |
| Canada | A/equine/Guelph/G03-0250/2003 | EU855743 |
| Canada | A/equine/Guelph/G03-55399/2003 | EU855741 |
| Canada | A/equine/Guelph/G04-54701/2004 | EU855742 |
| China | A/equine/Heilongjiang/1/2010 | JQ265982 |
| China | A/equine/Heilongjiang/10/2008 | EU794511 |
| China | A/equine/Heilongjiang/SS1/2013 | KC986390 |
| Japan | A/equine/Hokkaido/I828/2008 | AB543705 |
| Hong Kong | A/equine/Hong Kong/J/1992 | L27597 |
| China | A/equine/Huabei/1/2007 | GU571144 |
| China | A/equine/Hubei/6/2008 | EU794503 |
| Nigeria | A/equine/Ibadan/6/91 | X95637 |
| Nigeria | A/equine/Ibadan/9/91 | X95638 |
| USA | A/equine/Idaho/37875/1991 | CY018869 |
| China | A/equine/Inner Mongolia/8/2008 | EU794527 |
| Italy | A/equine/Italy/1062/1991 | CY032373 |
| Italy | A/equine/Italy/1199/1992 | CY032341 |
| Italy | A/equine/Italy/824/1991 | CY032333 |
| South Africa | A/equine/Johannesburg/1/1986 | CY032953 |
| Japan | A/equine/Kanazawa/1/2007 | AB369862 |
| India | A/equine/Katra-Jammu/6/2008 | FJ888344 |
| USA | A/equine/Kentucky/1/1978 | CY028796 |
| USA | A/equine/Kentucky/1/1981 | CY028828 |
| USA | A/equine/Kentucky/1/1986 | CY030093 |
| USA | A/equine/Kentucky/1/1987 | CY030101 |
| USA | A/equine/Kentucky/1/1990 | L39915 |
| USA | A/equine/Kentucky/1/1991 | CY030173 |
| USA | A/equine/Kentucky/1/1992 | CY030149 |
| USA | A/equine/Kentucky/1/1992 | L39917 |
| USA | A/equine/Kentucky/1/1994 | L39914 |
| USA | A/equine/Kentucky/1277/1990 | CY030133 |
| USA | A/equine/Kentucky/2/1980 | CY032937 |
| USA | A/equine/Kentucky/2/1981 | CY028820 |
| USA | A/equine/Kentucky/2/1986 | CY032221 |
| USA | A/equine/Kentucky/2/1987 | CY031538 |
| USA | A/equine/Kentucky/3/1981 | CY030077 |
| USA | A/equine/Kentucky/3/1986 | CY032229 |
| USA | A/equine/Kentucky/4/1980 | CY028804 |
| USA | A/equine/Kentucky/5/02 | AY855341 |
| USA | A/equine/Kentucky/692/1988 | CY030109 |
| USA | A/equine/Kentucky/694/1988 | CY030117 |
| USA | A/equine/Kentucky/698/1988 | CY030125 |
| USA | A/equine/Kentucky/8/1994 | CY030181 |
| USA | A/equine/Kentucky/bitter_boredom5/1976 | CY028852 |
| USA | A/equine/Kentucky/magnificent_genius1/1981 | CY028900 |
| USA | A/equine/Kentucky/pass_the_pepper1/1976 | CY028844 |
| USA | A/equine/Kentucky/Rosie100/1981 | CY030743 |
| Ireland | A/equine/Kildare/1/2007 | JN222936 |
| South Korea | A/equine/Kyonggi/SA1/2011 | JX844146 |
| China | A/equine/Liaoning/9/2008 | EU794519 |
| United Kingdom | A/equine/Lincolnshire/1/2007 | FJ195398 |
| Chile | A/equine/Lonquen/1/2006 | EU926631 |
| USA | A/equine/Massachussetts/213/2003 | DQ124193.1 |
| USA | A/equine/Miami/1963 | M29257 |
| Mongolia | A/equine/Mongolia/20/2011 | JX549063 |
| Mongolia | A/equine/Mongolia/6/2011 | AB745622 |
| USA | A/equine/Montana/9233/2007 | [CY067510](http://www.ncbi.nlm.nih.gov/entrez/viewer.fcgi??db=nucleotide&val=CY067510) |
| India | A/equine/Mysore/1/2008 | GU396101 |
| USA | A/equine/New Market/1/1979 | CY096891 |
| USA | A/equine/New Market/nasalwash1/1979 | CY096899 |
| USA | A/equine/New York/1/1975 | CY030189 |
| USA | A/equine/New York/1/1999 | DQ124189.1 |
| USA | A/equine/New York/146066/2007 | [CY067566](http://www.ncbi.nlm.nih.gov/entrez/viewer.fcgi??db=nucleotide&val=CY067566) |
| USA | A/equine/New York/452/2003 | [DQ124194](http://www.ncbi.nlm.nih.gov/entrez/viewer.fcgi??db=nucleotide&val=DQ124194) |
| USA | A/equine/New York/VR-297/1983 | CY028916 |
| United Kingdom | A/equine/Newmarket/5/2003 | FJ375213 |
| United Kingdom | A/Equine/Newmarket/76 | [M24722](http://www.ncbi.nlm.nih.gov/entrez/viewer.fcgi??db=nucleotide&val=M24722) |
| USA | A/equine/Ohio/1/2003 | DQ124192.1 |
| USA | A/equine/Ohio/113461-1/2005 | [CY067318](http://www.ncbi.nlm.nih.gov/entrez/viewer.fcgi??db=nucleotide&val=CY067318) |
| USA | A/equine/Ohio/113461-2/2005 | [CY067310](http://www.ncbi.nlm.nih.gov/entrez/viewer.fcgi??db=nucleotide&val=CY067310) |
| USA | A/equine/Ohio/113461-3/2005 | [CY067326](http://www.ncbi.nlm.nih.gov/entrez/viewer.fcgi??db=nucleotide&val=CY067326) |
| USA | A/equine/ORegon/78356/2012 | KM359812 |
| Kazakhstan | A/equine/Otar/764/2007 | JF683499 |
| China | A/equine/Qinghai/1/1994 | EU794535 |
| United Kingdom | A/equine/Richmond/1/2007 | FJ195395 |
| Italy | A/equine/Roma/5/1991 | CY032365 |
| Romania | A/equine/Romania/1/1980 | CY032389 |
| United Kingdom | A/equine/Rook/93753/1989 | CY032325 |
| Japan | A/equine/Sachiyama/1/1971 | CY034934 |
| USA | A/equine/Santa Fe/1/1985 | CY032309 |
| Chile | A/equine/Santiago/1/1985 | AY383755 |
| Brazil | A/equine/Sao Paulo/1/1969 | CY032397 |
| Brazil | A/equine/Sao Paulo/6/1963 | CY032293 |
| Kazakhstan | A/equine/South Kazakhstan/236/2012 | KF712451 |
| Spain | A/equine/Spain/1/2007 | [CY075851](http://www.ncbi.nlm.nih.gov/entrez/viewer.fcgi??db=nucleotide&val=CY075851) |
| United Kingdom | A/equine/Suffolk/89 | X68437 |
| United Kingdom | A/equine/Sussex/1/1989 | CY032317 |
| Switzerland | A/equine/Switzerland/1118/1979 | CY032381 |
| Switzerland | A/equine/Switzerland/173/1993 | CY032357 |
| Sweden | A/equine/Taby/1991 | S64310 |
| USA | A/equine/Tennessee/5/1985 | M24726 |
| USA | A/equine/Tennessee/5/1986 | CY030165 |
| USA | A/equine/Texas/117793/2005 | [CY067302](http://www.ncbi.nlm.nih.gov/entrez/viewer.fcgi??db=nucleotide&val=CY067302) |
| USA | A/equine/Texas/39655/1991 | CY030141 |
| Algeria | A/equine/Tiaret/1/2011 | KF317697 |
| Japan | A/equine/Tokyo/2/1971 | CY096915 |
| Japan | A/equine/Tottori/1/07 | AB591842 |
| Uruguay | A/equine/Uruguay/1/1963 | CY032421 |
| USA | A/equine/Virginia/131054-3/2005 | [CY067558](http://www.ncbi.nlm.nih.gov/entrez/viewer.fcgi??db=nucleotide&val=CY067558) |
| USA | A/equine/Wisconsin/1/03 | DQ222913 |
| China | A/equine/Xinjiang/1/2007 | EU794543 |
| China | A/equine/Xinjiang/2/2007 | EU794551 |
| China | A/equine/Xinjiang/3/2007 | EU794559 |
| China | A/equine/Xinjiang/4/2007 | EU794567 |
| China | A/equine/Xuzhou/01/2013 | KF806985 |
| Japan | A/equine/Yokohama/aq13/2010 | AB618504 |
| Japan | A/equine/Yokohama/aq19/2009 | AB544410 |
| Japan | A/equine/Yokohama/aq29/2011 | AB727557 |
| Japan | A/equine/Yokohama/aq5/2011 | AB727555 |
| Japan | A/equine/Yokohama/aq53/2011 | AB727559 |
| Japan | A/equine/Yokohama/aq79/2011 | AB727561 |
| Brazil | A/equine/Rio Grande do Sul/1/12 * | EPI584295 |
| Chile | A/equine/Colina/2/2012** | JX041592 |

* Nucleotide sequence obtained from the GISAID EpiFlu™ Database; ** A/equine/Colina/2/2012 was included to form the Partial HA1 data set.
